# Assessing Peripheral Blood Biomarkers and Predictive Patterns in Multiple Sclerosis Using Cytokines and Immune Gene Expression Profiles in Ocrelizumab-Treated Patients: Tracking Tumor Necrosis Factor

**DOI:** 10.3390/ijms262311295

**Published:** 2025-11-22

**Authors:** Bojan Jevtić, Nikola Momcilovic, Goran Stegnjaić, Milica Lazarević, Suzana Stanisavljević, Olivera Tamas, Nikola Veselinovic, Maja Budimkic, Sarlota Mesaros, Đorđe Miljković, Tatjana Pekmezovic, Jelena Drulovic, Neda Nikolovski

**Affiliations:** 1 Department of Immunology, Institute for Biological Research “Siniša Stanković”-National Institute of the Republic of Serbia, University of Belgrade, 11060 Belgrade, Serbia; bojan.jevtic@ibiss.bg.ac.rs (B.J.); goran.stegnjaic@ibiss.bg.ac.rs (G.S.); milica.lazarevic@ibiss.bg.ac.rs (M.L.); ssuzana@ibiss.bg.ac.rs (S.S.); djordjem@ibiss.bg.ac.rs (Đ.M.); 2 Clinic of Neurology, University Clinical Centre of Serbia, 11000 Belgrade, Serbia; nikolamom1993@gmail.com (N.M.); stojiljkovic.olivera@gmail.com (O.T.); n.veselinovich@gmail.com (N.V.); budim17@gmail.com (M.B.); sharlotam@gmail.com (S.M.); 3 Faculty of Medicine, University of Belgrade, 11000 Belgrade, Serbia; pekmezovic@sezampro.rs; 4 Institute of Epidemiology, University of Belgrade, 11000 Belgrade, Serbia

**Keywords:** multiple sclerosis, ocrelizumab, *TNF*, biomarker, B cells

## Abstract

Ocrelizumab, a humanized monoclonal anti-CD20 and B cell-depleting antibody, is a disease-modifying therapy for multiple sclerosis (MS), a chronic inflammatory, demyelinating, and neurodegenerative disease of the central nervous system. However, reliable predictive biomarkers of ocrelizumab’s effectiveness, such as cytokine expression profiles in peripheral blood mononuclear cells (PBMCs), are lacking. The aim of this study was to identify immunological biomarkers of ocrelizumab treatment response in MS patients, during a two-year follow-up. mRNA expression for specific immune molecules in PBMCs was measured, and consequently correlated with the clinical and radiological parameters of disease activity. PBMCs were obtained from 80 MS patients (35 with relapsing–remitting MS-RRMS and 45 with primary progressive-PPMS), immediately before initiating ocrelizumab treatment and thereafter every 6 months (before the administration of the next dose of ocrelizumab). Expression of the B cell marker *CD19*; the pro-inflammatory cytokines interleukin (*IL*)*1B*, *IL6*, and tumor necrosis factor (*TNF*); and a costimulatory cell marker *CD86* were determined. In both RMS and PPMS patients treated with ocrelizumab, higher baseline expression of *TNF* was statistically significantly associated with an increased risk of developing evidence of disease activity and a greater likelihood of disability progression, at month 24. This result implies that PBMCs’ *TNF* mRNA expression might be potentially considered as a prognostic biomarker of ocrelizumab effectiveness in MS patients. However, further studies comprising large cohorts and additional immunological parameters are warranted.

## 1. Introduction

Multiple sclerosis (MS) is a chronic inflammatory, demyelinating, and neurodegenerative disease of the central nervous system (CNS) that clinically begins with relapses (relapsing–remitting MS, RRMS) or with a steady deterioration of the neurological disability from onset (primary progressive MS, PPMS) [1,2]. Increasing evidence supports early intervention with high-efficacy disease-modifying therapies (DMTs), especially anti-cluster of differentiation 20 (CD20) drugs [3,4,5,6,7]. The choice of disease-modifying therapy (DMT) in MS depends on multiple factors, including patient characteristics (such as demographics, clinical and radiological features, comorbidities, and lifestyle factors); current treatment guidelines [8]; and patient preferences. Additionally, access to certain DMTs may be limited by regulatory or reimbursement restrictions. It has to be emphasized that safety considerations may also play a significant role in treatment selection [9].

For many years, MS was considered a primarily T cell-mediated disease [10,11]. However, in the last 10–15 years, evidence supporting the involvement of B cells in the pathogenesis of MS has begun to arise [12,13,14,15]. Clinical efficacy supports the importance of the role of B cells in the pathophysiology of MS, which has been, until now, well documented, both in the periphery and in the CNS [16,17,18]. Anti-CD20 monoclonal antibodies deplete B cells through several mechanisms, primarily antibody-dependent cellular cytotoxicity (ADCC), complement-dependent cytotoxicity (CDC), and antibody-dependent cellular phagocytosis (ADCP) [19]. Following ocrelizumab infusion, CD20 B cells are rapidly depleted in a matter of hours, mainly in the liver [20]. B cell repopulation begins in the bone marrow and spleen and, subsequently, in the blood, with memory and CD19 cells recovering at different rates [12]. Since CD19 expression largely overlaps with CD20, it is typically used to monitor B cell counts [12]. B cell reappearance can be defined when CD19 B cells comprise 1–2% of lymphocytes [21]. B cell-depleting anti-CD20 DMTs, such as ocrelizumab, represent highly effective drugs for reducing the relapse rate, magnetic resonance imaging (MRI) activity, and progression of the disease [22,23].

Ocrelizumab is a humanized monoclonal anti-CD20 antibody, designed to bind to the CD20 molecule, thus leading to the rapid depletion of B cells. Based on the successful results in the OPERA I and II study for RMS [10], and ORATORIO study for PPMS [24], ocrelizumab was approved for MS treatment by the FDA in 2017, and by the EMA in 2018 [25]. In the OPERA I and II trials, ocrelizumab showed its superiority in patients with RRMS compared to IFNβ-1a in reducing the annualized relapse rate (ARR), confirmed disability progression (CDP), and MRI activity after 96 weeks [10]. Results published from the extension phase III trials, OPERA I and II, provide evidence that longitudinal treatment with ocrelizumab, especially when started in the early stage of the disease, is safe and beneficial for patients with RRMS [26]. In the ORATORIO phase III trial, ocrelizumab reduced CDP in PPMS [24]. In ORATORIO, a phase III trial of ocrelizumab in PPMS patients versus placebo, the proportion of patients with 12-week and 16-week confirmed disability progression in the ocrelizumab arm was 32.9% and 29.6%, respectively, compared to 39.3% and 35.7% in the placebo arm [24].

It has been currently widely accepted that high-efficacy DMTs should be used early in MS treatment [27]. Patients with MS who initiated treatment with high-efficacy DMTs have improved health outcomes with lower ARR and disability progression for more than 10 years following treatment initiation, in comparison with those who began treatment with moderately effective DMTs [7]. With the introduction of highly effective MS treatments, the aim of achieving no evidence of disease activity (NEDA) has been established as a desirable outcome [28].

The aim of this study was to longitudinally investigate a real-world single-center cohort of RRMS and PPMS patients, followed for two years, after ocrelizumab initiation. Thus, expression of mRNA for specific immune molecules in the PBMCs of MS patients treated with ocrelizumab was measured, and correlated with the clinical brain MRI parameters over a 2-year follow-up period. The expression of a B cell marker (*CD19*), pro-inflammatory cytokines (*IL1B*, *IL6*, and *TNF*), and *CD86* (a costimulatory cell marker) in the PBMCs of 80 MS patients and 34 matched healthy controls was analyzed. Expression of these immune parameters was determined in PBMCs at the baseline (on the day of the first ocrelizumab dose) and subsequently every six months prior to the next infusion.

## 2. Results

Demographic and baseline clinical characteristics of the study population, separately for RRMS and PPMS, are presented in Table 1. A total of 80 MS patients, with a predominance of females, were followed for two years, from 2022 to 2024, during treatment with ocrelizumab. Baseline values obtained from MS patients were compared to the healthy control group, which was adjusted to the MS group at baseline, in both age and gender distribution.

**Figure 1:**
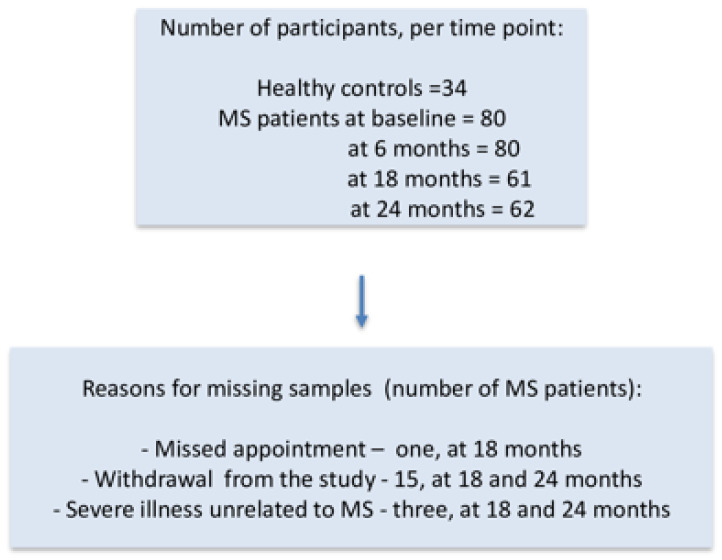
A flowchart presenting the number of participants from whom blood samples were collected at predefined time points and the reason for missing samples.

Peripheral blood samples were collected from MS patients at baseline (on the day of the first ocrelizumab dose) and subsequently every six months prior to the next infusion, over a two-year period. Samples from the healthy subjects were obtained once. Expression of the B cell marker *CD19*; costimulatory marker *CD86*; and the pro-inflammatory cytokines *IL1B*, *IL6*, and *TNF* in PBMCs was determined in PBMCs at the indicated time points. A flowchart with the number of participants from whom the blood samples were collected at predefined time points and the reasons for missing samples is presented in Figure 1. Data from all MS patients with missing samples were included in the analyses until they left the study.

**Figure 2:**
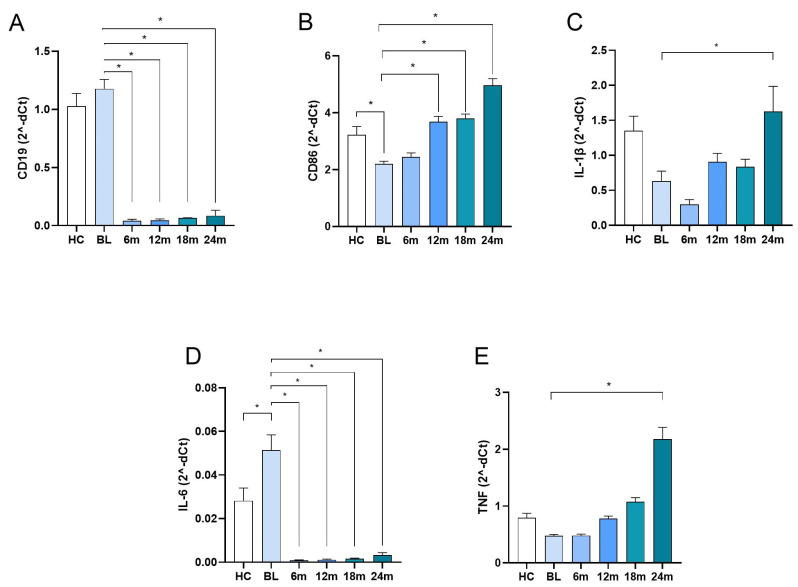
mRNA expression of *CD19*, *CD86*, *IL1B*, *IL6*, and *TNF* in PBMCs. Peripheral blood samples from MS patients were obtained at baseline (the day of the first ocrelizumab administration) and at 6-month intervals prior to each subsequent infusion over a two-year period. Samples from healthy controls were collected at a single time point. mRNA expression of (**A**) *CD19*; (**B**) *CD86*; (**C**) *IL1B*; (**D**) *IL6*; and (**E**) *TNF* was determined by real-time qPCR in PBMCs. Mean and SEM values are shown; * *p* < 0.05, significance is shown relative to baseline. Comparisons of values between groups were assessed using ANOVA with Bonferroni adjustment. HC—healthy control; BL—baseline.

*CD19*, a well-established marker of B lymphocytes, was used as an indicator of ocrelizumab treatment efficacy. There was no difference in the level of *CD19* expression between the HC and MS group at the baseline (Figure 2A). Ocrelizumab treatment led to a marked reduction in *CD19* expression, already at the first time point, and its expression remained extremely low throughout the observation period (Figure 2A), thus confirming sustained B cell depletion.

Expression of *CD86* in the MS group, at the baseline, was statistically significantly lower in comparison to the HC group (Figure 2B). Ocrelizumab treatment led to a gradual increase in *CD86* expression levels, with values obtained at months 12, 18, and 24 being statistically significantly higher in comparison to baseline values (Figure 2B). *IL1B* expression was also lower at baseline in MS patients relative to the HC, but without statistical significance. Also, there were no statistically significant differences in the values obtained after ocrelizumab treatment initiation in comparison to baseline values, except for samples obtained at 24 months, where a marked increase was observed (Figure 2C).

*IL6* expression levels were statistically significantly elevated at baseline in MS patients compared to the HC. Ocrelizumab treatment led to a strong reduction in *IL6* expression at all subsequent time points compared to baseline (Figure 2D).

*TNF* expression was without a statistically significant difference and between the values of MS patients at baseline and the HC (Figure 2E). Also, levels obtained after ocrelizumab treatment initiation were not statistically significantly different to baseline levels, except at month 24 (Figure 2E).

Comparisons of the gene expression levels in patients with MS treated with ocrelizumab, during a 2-year follow-up period, at different time points, and healthy controls, are provided in Table 2.

Furthermore, we compared the gene expression levels in RRMS and PPMS patients. No significant differences were found between the two groups for any of the parameters examined (Table 3).

Data related to the clinical activity of disease (EDSS, relapses) and MRI lesions during 2-year treatment with ocrelizumab in patients with RRMS and PPMS are presented in Table 4.

Finally, logistic regression analysis adjusted by sex, age, and baseline MRI activity was performed. Results of the analysis demonstrated that the only predictor for EDA was the expression of *TNF* in MS patients at baseline (Table 5), implicating that increased baseline expression of *TNF* was associated with higher probability of developing EDA during ocrelizumab treatment. Increased expression of *TNF* was also associated with greater odds of disability worsening, but neither with relapse activity nor with unique active lesions (Table 5). The odds ratio of the presence of combined unique active lesions and the presence of new or enlarging T2 lesions was associated with the increased expression of *IL1B* at baseline. None of the remaining immune parameters tested were significant predictors for EDA, after a 2-year follow-up.

Our findings demonstrate altered expression of CD86 and IL6 but not of CD19, ILB, and TNF in MS PBMCs compared to HC. Furthermore, elevated baseline TNF expression in persons with MS was associated with a higher probability of developing EDA and an increased risk of disability progression over a two-year follow-up in ocrelizumab-treated patients in both RRMS and PPMS.

## 3. Discussion

In our study, logistic regression analysis of the examined parameters showed that higher baseline expression of *TNF* was associated with an increased likelihood of developing EDA and a greater risk of disability progression over a 2-year follow-up in patients with both RRMS and PPMS treated with ocrelizumab. Ocrelizumab treatment significantly reduced expression of *CD19* and *IL6*, in patients with MS, after two years of treatment with ocrelizumab. During the same time period, the levels of *CD86*, *IL1B*, and *TNF* expression increased in this patient cohort.

We have demonstrated that, at baseline, MS patients had significantly lower levels of expression of *CD86* and significantly higher levels of *IL6* in PBMCs compared to HC. At the same time, there was no significant difference in the expression of *CD19, ILB,* and *TNF*. As a costimulatory molecule, expressed on the antigen-presenting cells, *CD86* has a role in activating T cells [29,30,31]. While there are studies showing that *CD86* is overexpressed in PBMCs at all stages of MS compared with the HC [32], to the best of our knowledge, there is no data showing how ocrelizumab affects expression of *CD86* in PBMCs of patients with MS. Its increased expression during the treatment can be explained as a compensation mechanism of other antigen-presenting cells, due to the B cell depletion. Patients suffering from MS have B cells that display various abnormalities, such as excessive production of pro-inflammatory cytokines (TNF, lymphotoxin-α, IL-6, and granulocyte-macrophage colony-stimulating factor (GM-CSF)) [14,33] and a reduction in regulatory B cells (Breg) that produce IL-10 [34]. Also, it was shown that ocrelizumab reduces the number of naive and memory B cells [35] and specifically B cells that produce IL-6, IL-10, GM-CSF, and TNF. In our study, ocrelizumab also reduced the expression of *IL6* in patients’ PBMCs, but it led to the increased expression of *IL1B* and *TNF,* over time. This discrepancy in the results might be explained by the differences in the methodology. While we quantified the mRNA expression of the cytokines in PBMCs, flow cytometry was used to assess the B cell subpopulations and production of cytokines in these cells by the others. Moreover, their analysis was limited to the baseline and a single follow-up at 6 months post-treatment initiation, whereas our study included baseline and four additional time points at 6-month intervals. In their study, the CD19^+^ population, assessed six months after the first ocrelizumab dose, showed a marked reduction in B cells producing TNF, IL-6, GM-CSF, and IL-10 compared to the baseline, along with relative increases in GM-CSF- and IL-6-producing B cells. In contrast, in our study, *TNF* expression remained unchanged compared with the baseline after the first ocrelizumab dose but showed a significant increase after 24 months of continuous therapy. These discrepancies may be explained by the differences in sampling frequency between the studies. Longitudinal assessment in a study is crucial, as it captures dynamic immune changes that may be missed with sparse sampling. These findings underscore the value of multi-time-point monitoring in providing a more comprehensive understanding of immune modulation over the course of treatment. The unusual increase in *IL1B* and *TNF* expression may be due to uneven recovery of immune cell types over time, or because B cell depletion alone does not fully halt pro-inflammatory signaling.

In our study, it has been demonstrated that the higher level of *TNF* expression, at baseline, was a predictor of an unfavorable outcome of ocrelizumab treatment. TNF is a cytokine that plays a pivotal role in regulating immune responses and inflammation. This molecule acts by binding to its receptors, TNFR1 and TNFR2, initiating signaling pathways that can lead to inflammation, cell survival, or cell death. While TNF is essential for host defense and tissue homeostasis, its dysregulated or excessive production is implicated in the pathogenesis of various inflammatory and autoimmune diseases [36] and also in MS within the CNS [37]. It plays a complex and multi-layered role in the immunopathogenesis of MS, as evidenced by findings from both preclinical models and human studies—including the unexpected worsening of disease observed in a clinical trial, non-selectively targeting TNF inhibition [38,39,40]. TNF is present in the cerebrospinal fluid of MS patients, and it is expressed by astrocytes, microglia, and endothelial cells within both acute and chronic active lesions in the MS brain [37,41,42,43]. It should be emphasized that high levels of soluble TNFR1 and low levels of soluble TNFR2 were identified as promising prognostic markers of disease progression in PPMS [44], while there are no available results regarding RRMS. This could reflect that the PPMS phenotype is less dependent on B cell-mediated mechanisms and more driven by persistent activation of innate immune pathways, particularly involving monocytes and microglia, which are well-known sources of TNF [45,46]. However, in our study, higher baseline TNF levels in MS patients likely indicate that the patients already have a heightened inflammatory state, even before treatment begins. In these individuals, the underlying disease processes may rely less on B cell-mediated mechanisms and more on broader inflammatory pathways. As a result, they may be less likely to benefit from B cell-depleting therapies such as ocrelizumab.

This observation is consistent with the possibility that, in a subset of patients, MS pathophysiology may be driven predominantly by TNF-mediated innate immune mechanisms that are not adequately targeted by anti-CD20 therapy, as implicated in the pivotal immunopathological studies [47]. Identifying such patients before the treatment initiation could help guide personalized therapeutic strategies such as alternative or combination therapies. Indeed, as therapeutic options for MS patients continue to expand, there is a growing need for biomarkers that can indicate how well a patient is responding to a particular therapy [48,49,50,51,52]. Being able to track treatment efficacy in a precise and timely manner helps clinicians make decisions that are in the best interest of each patient—whether that means continuing an effective therapy or switching to an alternative when benefits are limited. The ultimate goal is to identify biomarkers that can predict inadequacy of a certain therapy even before its start. This approach not only supports better long-term outcomes and quality of life for people living with MS, but also ensures that expensive disease-modifying therapies are used responsibly, contributing to a more balanced and sustainable healthcare system.

Our study has certain limitations. A larger number of study participants, longer follow-up period, and higher number of relevant molecules tested would contribute to more comprehensive findings, which could potentially contribute to the improvement of the treatment strategy in MS. Additionally, the focus of our study was to investigate transcriptional regulation and early immune activation; therefore, analysis at the mRNA level was performed as most appropriate. Changes in gene expression often occur prior to detectable differences in protein abundance, especially for cytokines, and assessing mRNA expression thus enables the detection of early regulatory events that may not yet be reflected at the protein level. However, protein expression analysis will be the aim of our further research in order to complement our findings.

## 4. Materials and Methods

PBMCs’ gene expression was monitored in ocrelizumab-treated MS patients and compared with clinical and MRI measures over 24 months, with blood collected at baseline and before each treatment cycle, i.e., every six months.

### 4.1. Patients

We recruited for this prospective study 80 patients with MS, who were followed for two years, from 2022 to 2024, during treatment with ocrelizumab. In this study, MS patients treated with ocrelizumab, as per Section 4.4 of the “ocrelizumab, Summary of product characteristics (SmPC)” [53] followed-up at the Clinic of Neurology, University Clinical Center of Serbia in Belgrade, were invited to participate. Blood samples were obtained at baseline, before the first administration of ocrelizumab, and thereafter every 6 months before the administration of the drug up to 24 months. Blood samples were also obtained from 34 age- and gender-matched healthy control (HC) subjects. The HC group was recruited among unaffected relatives and caregivers of MS patients. Laboratory data were prospectively collected after signed informed consent had been obtained from all study participants, with approval from the Ethics Committee of the Faculty of Medicine University of Belgrade (No. 1322/IX-65, 30 September 2021). Clinical data including Expanded Disability Status Scale (EDSS) score [54] were obtained from experienced MS clinicians at baseline, and at each 6-month follow-up. Inclusion criteria were the following: age ≥ 18 years, diagnosis of MS based on the 2017 McDonald criteria [55], and availability of brain MRI scans acquired longitudinally before and each year during ocrelizumab treatment during a 2-year follow-up.

The primary clinical outcome was evidence of disease activity (EDA) over 2 years of follow-up, defined as confirmed disability worsening (measured using the Expanded Disability Status Scale [EDSS]; score ranges from 0 to 10, with higher scores indicating higher levels of disability), relapse activity, or any new or enlarging lesions on MRI (cumulative unique active lesions) [56]. Secondary outcomes included each of these components individually.

### 4.2. Isolation of PBMCs

Human PBMCs were isolated from fresh blood samples obtained from MS patients and healthy individuals 4–6 h after blood collection. Blood specimens from patients were obtained for 2 years, starting before initiating ocrelizumab treatment and thereafter every 6 months, before next dosing. All procedures related to the use of human blood are in conformity with the recommendation provided in the Code of Ethics of the World Medical Association (Declaration of Helsinki). The blood samples were collected in EDTA vacutainer tubes (Improve, Guangzhou, China). The samples were diluted two times with PBS and overlaid on Histopaque 1077 (Sigma-Aldrich, St. Louis, MO, USA), at 720 g for 20 min without brake and acceleration. Afterwards, PBMCs were collected from the interlayer ring.

### 4.3. RNA Extraction and RT-qPCR Analysis

Total RNA was isolated from cells using Trizol reagent (Ambion, Foster City, CA, USA) and reverse transcribed using random hexamer primers and MMLV (Moloney murine leukemia virus) reverse transcriptase according to the manufacturer’s instructions (Fermentas, Vilnius, Lithuania), as previously described [57]. RNA quality and quantity were assessed using an Implen NanoPhotometer N60 (Munich, Germany). All samples met RNA purity criteria, with A260/A280 ratios ranging from 1.8 to 2.0. Reactions were performed in two technical replicates using SYBR green as the reagent for detection. All samples were run in the same conditions: volume (10 μL), annealing temperature (60 °C), and cycle number (40 cycles). Any replicate with a Ct difference exceeding 0.5 was re-run to ensure reliability. The PCR primers (Metabion, Martinsried, Germany) were as follows: *ACT*: F: CAT GTA CGT TGC TAT CCA GGC; R: CTC CTT AAT GTC ACG CAC GAT; *CD19*: F: TCA GCT GTG ACT TTG GCT TAT CTG; R: AGT CAT TCG CTT TCT TTT CCT; *CD86*: F: CTG CTC ATC TAT ACA CGG TTA CC; R: GGA AAC GTC GTA CAG TTC TGT G; *IL1B*: F: ACA GAT GAA GTG CTC CTT CCA; R: GTC GGA GAT TCG TAG CTG GAT; *IL6*: F: GACAACTTTGGCATTGTGG; R: ATGCAGGGATGATGTTCTG; *TNF*: F: CCCTCACACTCAGATCATCTTCT; R: GCT ACG ACG TGG GCT ACA G. Primers were designed using Primer3Manager version 3.3.0 software. The accumulation of PCR products was detected in real time, and the results were analyzed with QuantStudio 3 software. Relative RNA expression is presented as 2^−dCt^, where dCt is the difference between the Ct values of a gene of interest and the endogenous control (β-actin).

### 4.4. Statistical Analysis

In the descriptive analysis, frequencies, proportions, and mean and median values, along with the standard deviation (SD) or standard error of measurement (SEM) (as indicated), and interquartile range, were used. Comparisons of values between groups were assessed using ANOVA with Bonferroni adjustment. To determine factors independently associated with EDA, logistic regression analysis adjusted by sex, age, and baseline MRI activity was performed, with OR (odds ratio) and 95% CI (confidence interval) as measures of effect. In this analysis, the dependent variable was the presence of EDA and the following gene expression of immune variables in PBMCs: *CD19*, *CD86*, *IL1B*, *IL6*, and *TNF* were used as independent factors. All these parameters are dichotomized as (1) lower or equal to and (2) greater than the median value: *CD19* = 0.9670, *CD86* = 2.0390, *IL1B* = 0.0700, *IL6* = 0.0230, and *TNF* = 0.449. Data were analyzed using The Statistical Package for Social Sciences (SPSS) version 20.0.

## 5. Conclusions

Our study highlights the potential importance of baseline *TNF* expression, as one of the factors in taking decisions regarding DMT selection, especially B cell-depleting anti-CD20 therapies, for MS patients. It also underscores the value of longitudinal studies, which allow a more precise interpretation of treatment-related immune changes. This reminds us of the importance of the heterogeneity of MS pathogenesis and emphasizes the need for a biomarker-guided therapeutic stratification. Although there are studies investigating ocrelizumab’s effect on the immune system, this remains an evolving field. Further research is needed to deepen our understanding of the mechanisms of action of ocrelizumab in MS and to potentially further enhance its therapeutic impact. Such insights may contribute to a more personalized approach to disease management, helping clinicians better predict which patients are more likely to benefit from specific therapeutic strategies. Ultimately, this could lead to improved clinical outcomes and a more targeted use of resources in the treatment of MS.

## Figures and Tables

**Table 1:** Demographic and clinical characteristics of MS patients.

Variables	RRMS (n = 35)	PPMS (n = 45)
Sex (number, %)		
-Male	11 (31.4%)	9 (20%)
-Female	24 (68.6%)	36 (80%)
Age at the beginning of the ocrelizumab treatment (years) (mean ± SD)	40.9 ± 8.7	44.7 ± 10.0
Duration of disease (years) (mean ± SD)	11.7 ± 7.2	8.6 ± 5.7
EDSS score at the beginning of the ocrelizumab treatment (median, IQR)	4.0 (2.5)	6.0 (2.0)
Relapses in the year prior to the ocrelizumab treatment (number, %)		
-Yes	30 (85.7%)	/
-No	5 (14.3%)	/
Active MRI lesions at the beginning of the ocrelizumab treatment (number, %)		
-Yes	10 (28.6%)	5 (11.1%)
-No	25 (71.4%)	40 (88.9%)

MS—multiple sclerosis; IQR—interquartile range; RR—relapsing–remitting; PP—primary progressive; EDSS—Expanded Disability Status Scale.

**Table 2:** Multiple comparisons of the gene expression levels in patients with MS treated with ocrelizumab, during a 2-year follow-up period, at different time points, and healthy controls.

Molecules	MS Patients Treated with Ocrelizumab					Healthy Controls	*p*
	Baseline	After 6 Months	After 12 Months	After 18 Months	After 24 Months		
*CD19*	1.177 ± 0.716	0.042 ± 0.107	0.044 ± 0.103	0.066 ± 0.016	0.085 ± 0.375	1.026 ± 0.649	<0.001 ^1^
*CD86*	2.199 ± 0.852	2.449 ± 1.237	3.686 ± 1.542	3.798 ± 1.206	4.959 ± 1.876	3.227 ± 1.656	<0.001 ^2^
*IL1B*	0.631 ± 1.225	0.298 ± 0.615	0.905 ± 0.998	0.834 ± 0.851	1.625 ± 2.838	1.353 ± 1.196	<0.001 ^3^
*IL6*	0.051 ± 0.063	0.001 ± 0.001	0.001 ± 0.295	0.002 ± 0.009	0.003 ± 0.368	0.028 ± 0.034	<0.001 ^1^
*TNF*	0.478 ± 0.203	0.480 ± 0.234	0.780 ± 0.365	1.0759 ± 0.560	2.178 ± 1.635	0.792 ± 0.472	<0.001 ^4^

CD—cluster of differentiation; IL—interleukin; TNF—tumor necrosis factor. ^1^ Healthy controls vs. Baseline; Baseline vs. after 6 months, 12 months, 18 months, and 24 months, respectively. ^2^ Baseline vs. after 12 months, 18 months, and 24 months, respectively, and healthy controls vs. baseline; *p* = 0.026. ^3^ Baseline vs. after 24 months. ^4^ Baseline vs. after 18 months and 24 months, respectively.

**Table 3:** Comparison of gene expression between RRMS and PPMS patients.

Variable	All Patients (Mean ± SD)	RRMS (Mean ± SD)	PPMS (Mean ± SD)	*p*
*CD19*				
BL	1.1170 ± 0.7163	1.2414 ± 0.9411	1.0200 ± 0.4637	0.172
after 6 months	0.0416 ± 0.1073	0.0399 ± 0.0726	0.0429 ± 0.1287	0.904
after 12 months	0.0440 ± 0.1031	0.0240 ± 0.4110	0.0589 ± 0.1302	0.193
after 18 months	0.0660 ± 0.1249	0.0389 ± 0.1016	0.0863 ± 0.1373	0.144
after 24 months	0.0851 ± 0.3745	0.0311 ± 0.0840	0.1296 ± 0.4990	0.307
*CD86*				
BL	2.1949 ± 0.8591	2.1770 ± 0.9535	2.2082 ± 0.7887	0.876
after 6 months	2.4486 ± 1.2369	2.3634 ± 1.1103	2.5147 ± 1.3356	0.591
after 12 months	3.6857 ± 1.5417	3.9643 ± 1.6941	3.4458 ± 1.3763	0.172
after 18 months	3.7978 ± 1.2063	3.4866 ± 0.9775	4.0290 ± 1.3177	0.082
after 24 months	4.9589 ± 2.7263	4.4361 ± 2.4722	5.3893 ± 2.8838	0.173
*IL1B*				
BL	0.6309 ± 1.2254	0.5105 ± 1.1409	0.7149 ± 1.2876	0.487
after 6 months	0.2980 ± 0.6145	0.3211 ± 0.6191	0.2800 ± 0.6174	0.769
after 12 months	0.9048 ± 0.9982	0.8922 ± 0.9611	0.9156 ± 1.0426	0.925
after 18 months	0.8341 ± 0.8518	0.8865 ± 0.9931	0.7951 ± 0.7426	0.682
after 24 months	1.6249 ± 2.8376	1.3832 ± 1.2565	1.8239 ± 3.6746	0.547
*IL6*				
BL	0.0514 ± 0.0629	0.0584 ± 0.0743	0.0459 ± 0.0526	0.382
after 6 months	0.0009 ± 0.0013	0.0011 ± 0.0015	0.0008 ± 0.0011	0.343
after 12 months	0.0011 ± 0.0023	0.0007 ± 0.0017	0.0015 ± 0.0027	0.187
after 18 months	0.0015 ± 0.0029	0.0014 ± 0.0032	0.0016 ± 0.0028	0.809
after 24 months	0.0032 ± 0.0093	0.0024 ± 0.0073	0.0039 ± 0.0107	0.52
*TNF*				
BL	0.4776 ± 0.2027	0.4563 ± 0.1802	0.4862 ± 0.2188	0.663
after 6 months	0.4801 ± 0.2338	0.4880 ± 0.2369	0.4741 ± 0.2339	0.793
after 12 months	0.7801 ± 0.3648	0.8124 ± 0.3861	0.7522 ± 0.3485	0.505
after 18 months	1.0749 ± 0.5595	1.0015 ± 0.4972	2.3645 ± 1.7958	0.381
after 24 months	2.1783 ± 1.6354	1.9522 ± 1.4157	2.3645 ± 1.7958	0.327

BL—baseline; RRMS—relapsing–remitting multiple sclerosis; PPMS—primary progressive multiple sclerosis; CD—cluster of differentiation; IL—interleukin; TNF—tumor necrosis factor.

**Table 4:** Disease activity during 2-year treatment with ocrelizumab in patients with RRMS and PPMS.

Variable	RRMS (n = 35)		PPMS (n = 45)	
	During First Year	During Second Year	During First Year	During Second Year
Patients with relapses (no, %)	2 (5.7%)	3 (9.1%)	/	/
EDSS (median, IQR)	4.0 (2.0)	4.0 (3.0)	6.0 (2.5)	6.0 (2.5)
Patients with active, Gd+ MRI lesions (no, %)	1 (2.9%)	1 (3.1%)	1 (2.4%)	1 (2.6%)
Patients with new/enlarging T2w MRI lesions (no, %)	3 (8.6%)	4 (11.4%)	2 (4.7%)	2 (5.3%)

MS—multiple sclerosis; IQR—interquartile range; RR—relapsing–remitting; PP—primary progressive; EDSS—Expanded Disability Status Scale; Gd+—gadolinium-enhancing.

**Table 5:** Logistic regression analysis for predictors of EDA.

	*IL1B*			*TNF*		
	OR	95% CI	*p*	OR	95% CI	*p*
Relapses	4.20	0.62–41.62	0.999	1.29	0.07–22.62	0.618
Disability progression	0.94	0.36–2.51	0.908	4.93	1.70–14.26	**0.003**
Gadolinium-enhancing lesions	4.89	0.45–35.23	0.315	1.08	0.32–3.72	0.900
New or enlarging T2 lesions	6.06	1.44–25.56	**0.014**	1.35	0.46–4.28	0.799
Combined unique active lesions	5.13	1.21–21.84	**0.027**	1.25	0.36–4.33	0.722
Evidence of disease activity	1.76	0.67–4.67	0.253	3.34	1.27–8.80	**0.015**

OR—odds ratio; 95% CI—confidence interval; *p*—probability; bolded values denote statistical significance.

## Data Availability

The original contributions presented in this study are included in the article. Further inquiries can be directed to the corresponding authors.

## Abbreviations

The following abbreviations are used in this manuscript:

MS	multiple sclerosis
RRMS	relapsing–remitting multiple sclerosis
PBMCs	peripheral blood mononuclear cells
IL	interleukin
TNF	tumor necrosis factor
EDA	evidence of disease activity
CNS	central nervous system
DMT	disease-modifying therapies
CD20	cluster of differentiation 20
MRI	magnetic resonance imaging
ARR	annualized relapse rate
CDP	confirmed disability progression
HC	healthy control
BL	baseline
EDSS	Expanded Disability Status Scale
SD	standard deviation
SEM	standard error of measurement
IQR	interquartile range
Breg	regulatory B cells
GM-CSF	granulocyte–macrophage colony-stimulating factor
